# Clinical relevance of tertiary lymphoid structures in esophageal squamous cell carcinoma

**DOI:** 10.1186/s12885-022-09777-w

**Published:** 2022-06-24

**Authors:** Sota Deguchi, Hiroaki Tanaka, Shugo Suzuki, Seji Natsuki, Takuya Mori, Yuichiro Miki, Mami Yoshii, Tatsuro Tamura, Takahiro Toyokawa, Shigeru Lee, Kazuya Muguruma, Hideki Wanibuchi, Masaichi Ohira

**Affiliations:** 1 Department of Gastroenterological Surgery, Osaka City University Graduate School of Medicine, 1-4-3, Asahimachi, Abenoku, Osaka, 545-8585 Japan; 2 Department of Molecular Pathology, Osaka City University Graduate School of Medicine, Osaka, Japan

**Keywords:** Esophageal squamous cell carcinoma, Tertiary lymphoid structures, B cell, Chemotherapy, Immunotherapy

## Abstract

**Background:**

Tertiary lymphoid structures (TLSs) have been reported to be involved in immune responses in many carcinomas. This study investigated the significance of TLSs in esophageal squamous cell carcinoma, focusing on TLS maturation.

**Methods:**

The relationships of TLSs with clinicopathological features of 236 patients who underwent curative surgery for stage 0-IV esophageal squamous cell carcinoma were investigated. Mature TLSs, in which the germinal center formation was rich in CD23^+^ cells, were classified as TLSs containing a germinal center (GC-TLSs). GC-TLS densities were measured, and CD8^+^ cells were counted. The prognostic impact of GC-TLSs was assessed by Kaplan–Meier plots using the log-rank test for the relapse-free survival. A comparative study of GC-TLSs was performed using the Wilcoxon rank sum test. The relationship between GC-TLSs and CD8^+^ cells was examined by Spearman’s rank correlation coefficient test.

**Results:**

TLSs were located mainly at the invasive margin of the tumor in cases with esophageal squamous cell carcinoma. Among the patients treated with neoadjuvant chemotherapy, those with advanced disease had a better prognosis in the GC-TLS high-density group than did those in the GC-TLS low-density group. Patients in whom neoadjuvant chemotherapy was effective had more GC-TLSs than those in whom it was less effective. The density of GC-TLSs and the number of tumor-infiltrating CD8^+^ cells were higher in patients treated with neoadjuvant chemotherapy than in those without chemotherapy, and a weak correlation between the density of GC-TLSs and the number of tumor-infiltrating CD8^+^ cells was observed. Moreover, co-culturing of PBMCs with an anticancer drug-treated esophageal squamous cell carcinoma cell line increased the CD20 and CD23 expression in PBMCs in vitro.

**Conclusion:**

TLS maturation may be important for evaluating the local tumor immune response in patients treated with neoadjuvant chemotherapy for esophageal squamous cell carcinoma. The present results suggest that TLS maturation may be a useful target for predicting the efficacy of immunotherapy, including immune checkpoint inhibitor treatment for esophageal squamous cell carcinoma.

## Introduction

Although esophageal cancer has a poor prognosis [1], recent clinical trials have demonstrated the efficacy of immune checkpoint inhibitors, and immunotherapy will undoubtedly become more important for esophageal cancer in the future. Tumor-infiltrating T cells play a key role in the immune response of the tumor microenvironment, which is crucial for the efficacy of immunotherapy. The function of T cells in the tumor microenvironment has been well studied, centering on cytotoxic T cells. On the other hand, research is underway on whether B cells have a strong tumor-promoting effect or an anti-tumor effect. Tertiary lymphoid structures (TLSs) are ectopic lymphoid tissue that are temporarily formed in the affected area, not the secondary lymphoid organs, and are mainly composed of follicular dendritic cells, B cell regions, T cell regions, and high endothelial venules (HEVs) [2]. It has recently been reported that TLSs are found in the microenvironment of tumors [3]. We have previously reported that the presence of TLSs around gastric cancer is associated with a good prognosis, that increased granzyme B and perforin expression is observed around TLSs, and that memory T cells are present around TLSs [4, 5]. Zhao et al. reported that TLS-rich is a prognostic factor for superficial esophageal cancer [6]. Ruffin et al. reported that B-cell function forming TLS and internal GCs is important in head and neck cancers that histologically resemble ESCC [7].

However, the significance of TLSs in esophageal squamous cell carcinoma (ESCC) has not been clarified. The reason for this is that the current standard of care for ESCC is based on multidisciplinary treatment combining chemotherapy, radiation therapy, and surgery, which eventually modifies the local immune environment. In order to identify TLSs that elicit anti-tumor immune responses, it may be important to examine the phenotype of TLSs. It has been reported in pancreatic cancer and other cancers that patients with more mature TLSs have a better prognosis [8]. It has recently been reported that a second follicular TLS with a germinal center is present in the invasive margin of the tumor [9].

In this study, the relationship between TLS formation and prognosis in ESCC, especially the clinical significance of TLS maturation in cases treated with neoadjuvant chemotherapy (NAC), was investigated.

## Materials and methods

### Clinical samples

Tumor samples were obtained from 293 patients (mean age, 66 years) with primary ESCC who underwent surgical resection at the Department of Gastroenterological Surgery, Osaka City University Hospital, between 2011 and 2014. In this study, the following cases were excluded: 7 patients undergone endoscopic submucosal dissection, 9 cases of non-radical resection, 31 patients of preoperative radiotherapy, 4 patients who died during the perioperative period, 4 patients with intramural metastasis in the stomach, and 2 patients with pathological complete response by NAC. A total of 236 patients (mean age, 67 years) who had undergone esophagectomy with reconstruction for ESCC were enrolled in this study. The median follow-up time was 51 months (range 0–100 months). Indications for preoperative chemotherapy and details of the regimens were based on the esophageal cancer treatment guidelines. The preoperative regimen was 5-FU + cisplatin (FP), 5-FU + nedaplatin (FGP), or 5-FU + cisplatin + docetaxel (DCF). The chemotherapy dose was reduced as needed according to patient condition and adverse events. None of the patients received immunotherapy prior to surgery. Tumors were diagnosed histologically based on the 11^th^ Edition of the Japanese Classification of Esophageal Cancer [10, 11]. Upper gastrointestinal endoscopy, CT scan and fluorodeoxyglucose-position emission tomography imaging were performed in all patients for clinical diagnosis. The diagnosis was based on the consensus of all esophageal surgeons in our hospital. Informed consents were obtained from all patients. The differentiation of squamous cell carcinoma was classified as follows: well-differentiated type (*n* = 20), moderately differentiated type (*n* = 118), poorly differentiated type (*n* = 87), and others (*n* = 11). Patients received adjuvant chemotherapy in case with pathologically diagnosed lymph node metastasis.

### Immunohistochemical of tissue sections

Formalin-fixed paraffin-embedded (FFPE) tumor blocks with representative tumor areas were collected from 236 consecutive patients with ESCC. Immunohistochemical staining was performed according to previous reports from our department [4]. In briefly, immunohistochemical staining was performed on 4-μm-thick sections of paraffin-embedded tumor blocks from ESCC patients. After incubation at 60 ˚C for 10 min, the sections were deparaffinized in xylene and rehydrated with a graded ethanol series (70%, 80%, 90% and 100%). Endogenous peroxidase activity was blocked using absolute methanol containing 3% hydrogen peroxidase for 15 min. After washing the sections in PBS, antigens were retrieved by microwave treatment. Nonspecific binding was subsequently blocked using nonspecific staining blocking reagent (DAKO, Kyoto, Japan). The sections were then reacted with the primary antibody listed below, standing at 4 ˚C overnight. All reactions were performed using appropriate positive and negative controls. Antibodies used for immunohistochemical analyses were as follows: a mouse monoclonal anti-CD3 antibody (clone: F7.2.38, 1:50, high pH retrieval; Abcam, Cambridge, UK), a mouse monoclonal anti-CD8 antibody (clone: F7.2.38, 1:100, high pH retrieval; Abcam, Cambridge, UK), a mouse monoclonal anti-CD20 antibody (clone: IR604, prediluted, low pH, Merck, Darmstadt, Germany), a mouse monoclonal anti-CD21 antibody (clone: 2G9, prediluted, low pH, Nichirei, Tokyo, Japan), a rabbit monoclonal anti-CD23 antibody (clone: SP23, prediluted, low pH, Nichirei, Tokyo, Japan), and a rat monoclonal anti-PNAd (clone: 120,801, 1:25, high pH, Nichirei, Tokyo, Japan). Sections were incubated with secondary antibody histofine reagent (Nichirei, Tokyo, Japan), and the signal was visualized using 3–3’-diaminobenzidine (DAB). Sections were counterstained with hematoxylin before mounting.

### Evaluation of immunohistochemistry

Tumor slides stained with anti-CD20 and anti-CD23 antibodies were scanned at low magnification to select three fields (9.1 mm^2^; original magnification 20 × ; 1600 × 1200 resolution) with the greatest number of intratumoral and peritumoral CD20^+^ or CD23^+^ cells. The microscopic images were imported from the digital photo filing system DP-73 (Olympus, Tokyo, Japan). The area (mm^2^) of CD20^+^ cells was measured, and the percentage area (%) of each field that was covered by the CD20^+^ or CD23^+^ area was calculated with Image J software (NIH, Bethesda, MD). For the CD20^+^ and CD23^+^ areas, clusters occupying ≥ 0.04% and ≥ 0.01%, respectively, were counted as positive. CD20 and CD23 densities were determined as the mean values of the percentage areas in the three fields. Tumor slides stained with anti-CD8 antibody were scanned at high magnification, and five fields (original magnification 400 ×) were selected with staining of intratumoral and peritumoral CD8^+^ cells. The mean numbers of positively immunostained cells per field were counted using Image J software. To determine the cutoff value, each histogram was generated, and the quartile was calculated. A histogram of the obtained numbers was created to set the cutoff values for CD20, CD23, and CD8, respectively. The cutoff value was set to 0.863 for the first quartile for CD20, 0.128 for the second quartile for CD23, and 64 for the first quartile for CD8.

### Survival analysis

The association with relapse-free survival (RFS) was analyzed initially by a Kaplan–Meier curve and the log-rank test. RFS curves were drawn using the Kaplan–Meier method, and the log-rank test was used to assess the significance of differences in survival. RFS was defined as the time between the day of surgery and recurrence. *P*-values < 0.05 were considered significant. Each statistical analysis was performed using the JMP software program (SAS Institute, Cary, NC, USA).

### Cell line

The “T.T” ESCC cell lines were used in this study. T.T human esophageal squamous cell carcinoma cells were obtained from the Health Science Research Resources Bank (Osaka, Japan). The cells were cultured at 37˚C and in 5% CO2. The media used were DMEM (FUJIFILM Wako Pure Chemical Corporation, Tokyo, Japan) and RPMI-1640 (FUJIFILM Wako Pure Chemical Corporation, Tokyo, Japan), supplemented with 10% fetal bovine serum (FBS; Gibco, NY, USA), 100 IU/ml penicillin (ICN Biomedicals, CA, USA), 100 mg/ml streptomycin (ICN Biomedicals, CA, USA), and 0.5 mM sodium pyruvate (Bioproducts).

### In vitro* cell co-culture model*

T.T cells (1 × 10^6^/ml) were plated in 10 ml of full medium treated with or without treatment with 5-FU and CDDP (30 µM) for 24 h. PBMCs were isolated from total 10 healthy donors using Ficoll density gradient centrifugation with Ficoll-Paque™ PLUS (Cytiva, Tokyo, Japan) at 1,025 × g for 30 min at 20 ˚C, with the brakes off. The PBMCs (1 × 10^7^/ml) were then co-cultured with naïve or chemical-treated T.T cells (1 × 10^6^/ml) for 24 h (*n* = 7 and 3, respectively). The ratio of CD20^+^ B cells, CD23^+^ B cells, and CD8^+^ T cells in the PBMCs after co-culture with T.T cells was examined by flow cytometry. In brief, after saturation with BD Fetal Bovine Serum Stain Buffer (BD Biosciences, NJ, USA), mononuclear cells were incubated with the primary antibodies and Fc-receptor blocking buffer (cat. no. 564220, 2% HS in PBS) for 30 min at 4 ˚C in the dark. The following monoclonal directly labeled anti-human antibodies were used: PE-labeled anti-CD20 (cat. no. 590961), BV421-labeled anti-CD23 (cat. no. 562707) and FITC- labeled anti-CD8 (cat. no. 555634). The evaluation of dead cells was performed with 7-Amino-Actinomycin D (cat. no. 559925). All antibodies used for flow cytometry were purchased from BD Biosciences. Flow cytometric analyses were performed with the BD LSRFortessa™ X-20 (BD Biosciences).

### Statistical analysis

Chi-square test and Fisher’s exact test were used to assess the associations of the expressions of CD20^+^ B cells, CD23^+^ B cells, and CD8^+^ T cells with clinicopathological features. The degree of infiltration between the density of CD20^+^/CD23^+^ B cells and the number of CD8^+^ T cells was compared by Spearman’s correlation coefficient. The regression line was determined by plotting the number of each cell, and the correlation was examined. A Cox proportional hazard model was used for univariate and multivariate analyses of prognostic factors. *P*-values < 0.05 were considered significant. Correlation strength was defined as weak (0.2 < *r* ≤ 0.4) and strong (*r* > 0.7). The Wilcoxon rank sum test was used to compare the expressions of CD20^+^ B cells, CD23^+^ B cells, and CD8^+^ T cells between the two groups.

## Results

### Relationships of TLSs with clinicopathological features

**Fig. 1:**
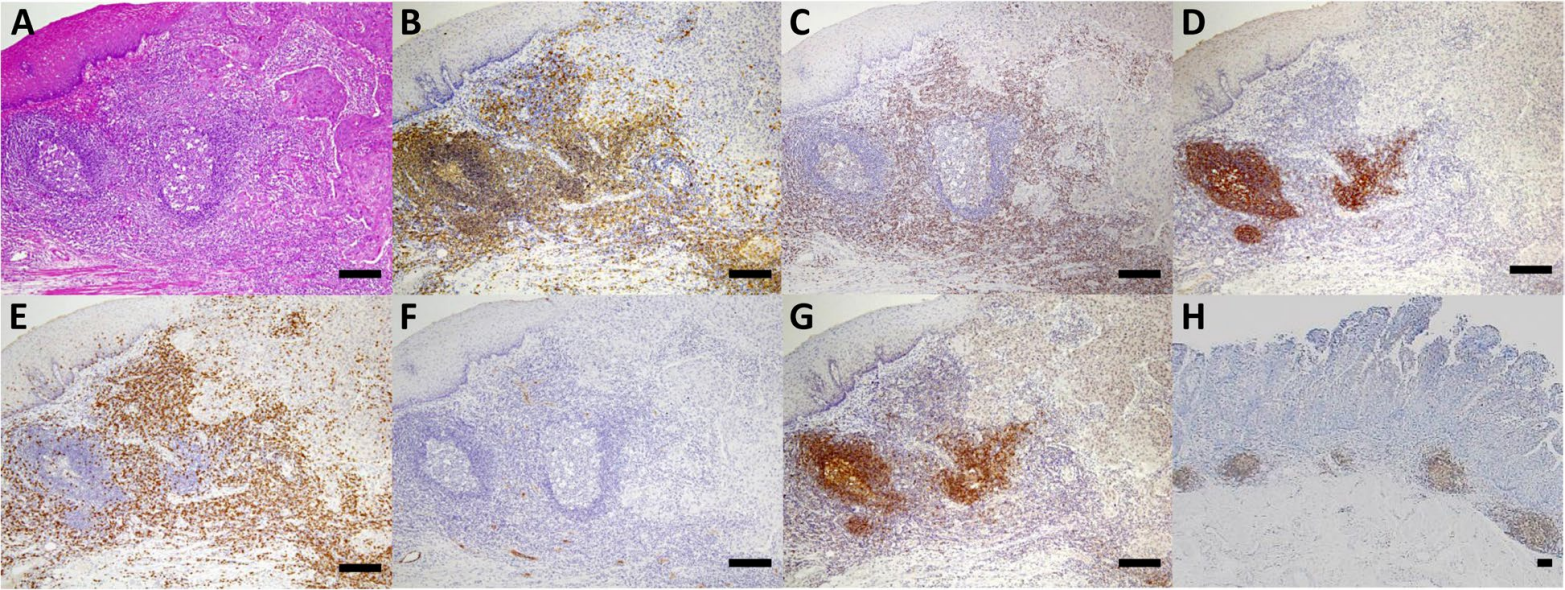
Tertiary lymphoid structures in ESCC. The pictures show representative immunohistochemistry findings on consecutive sections of the same ESCC tissue samples to identify TLSs. (A) Hematoxylin and eosin (HE) staining and immunohistochemical staining of (B, H) CD20, (C) CD3, (D) CD21, (E) CD8, (F) PNAd, and (G) CD23 in ESCC. Scale bars, 200 µm

**Fig. 2:**
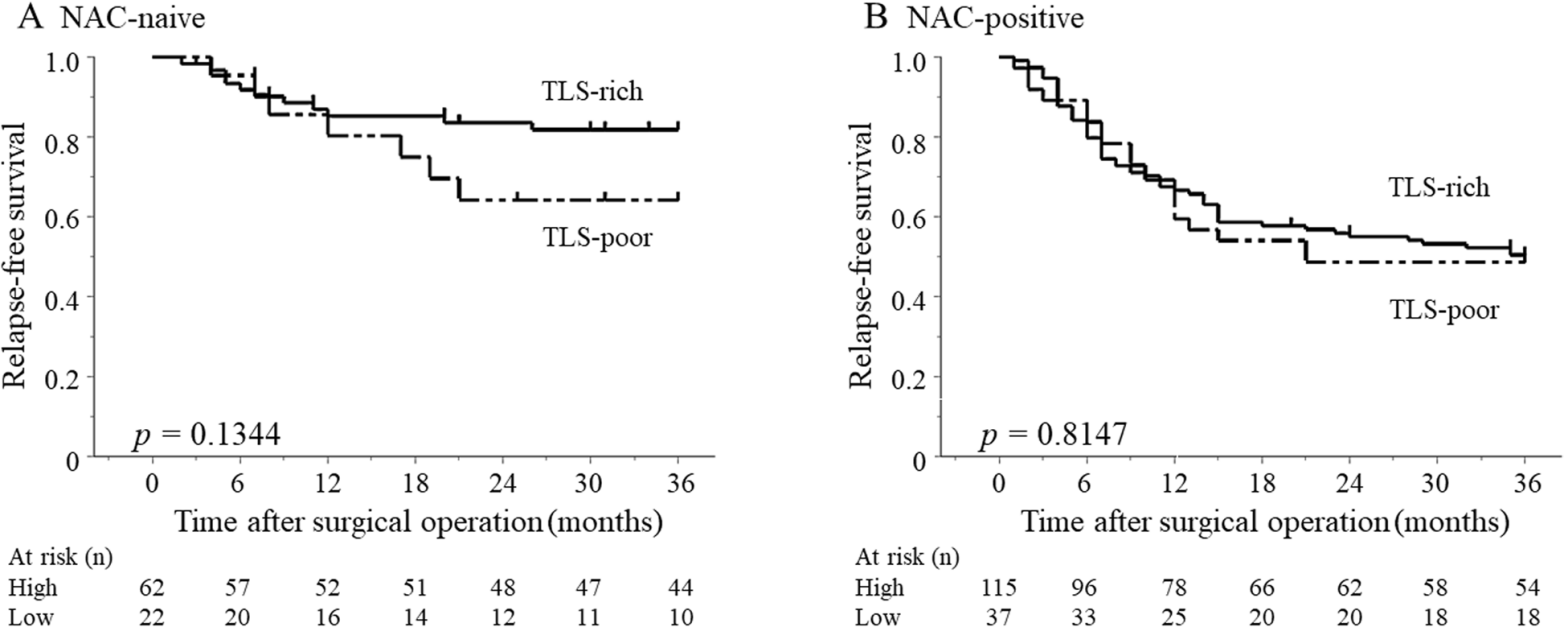
Kaplan–Meier curves of the relapse-free survival for CD20-TLS in patients with ESCC. A. The survival rate of patients in the TLS-rich group tended to be higher than that of patients in the TLS-poor group (survival rate at 36 months: 81.8%, and 64.2%, respectively). B There was no significant difference in the survival of the patients who received neoadjuvant chemotherapy in comparison to the TLS-rich group and the TLS-poor group (survival rate at 36 months: 50.5%, and 48.7%, respectively)

A representative stained slide is shown in Fig. 1. Peritumoral regions of clinical samples that were stained with HE were examined microscopically, and lymphocytes aggregating in the peritumoral regions were confirmed (Fig. 1A). Anti-CD20 staining showed positive cells at the same site (Fig. 1B). Regardless of whether CD20 expression was high or low, CD20^+^ B cells appeared mostly as aggregates that were located mainly at the invasive margin of the tumor and were present to line the tumor. The clusters of CD20^+^ B cells were surrounded by a CD3^+^ T cell rich area, and CD21^+^ FDCs formed a tight network in the B cell zone of the follicle (Fig. 1C, D). On anti-CD8 staining, CD8^+^ cells were observed outside aggregating CD20^+^ cells and inside tumoral areas as tumor infiltrating lymphocytes (Fig. 1E). The structure of HEV within the CD20-TLS was identified (Fig. 1F). Therefore, TLS was defined as CD20^+^ B cell aggregation, and its association with clinical outcomes was evaluated in this study. In the loupe image, TLSs were present to line the vicinity of the tumor (Fig. 1H). To examine the impact of tumor-associated TLSs on clinicopathological features, the patients were divided into two groups based on the density of CD20^+^ cells: a TLS-rich group (*n* = 177) and a TLS-poor group (*n* = 69). The relationship between the patient’ clinicopathological factors and each lymphocyte type is shown in Table 1. Young patients tended to have a lower TLS density than elderly patients (*p* = 0.055). Regarding TLS density, there was no difference between the two groups due to other factors. Patients were also classified into two groups based on CD8: a CD8-high group (*n* = 178) and a CD8-low group (*n* = 58). Regarding CD8 infiltration, there were significant differences in the sex ratios, clinical tumor depth, clinical nodal metastasis, clinical stage, with or without NAC, and pathological tumor depth. Kaplan–Meier survival analysis showed that survival tended to be better in the TLS-rich group than in the TLS-poor group in patients who had not received adjuvant chemotherapy (*p* = 0.1344, log-rank test) (Fig. 2A). In contrast, there was no significant difference in survival in the patients who received NAC (Fig. 2B).

**Table 1:** Correlations with the clinicopathological factors and density of the CD20^+^ B cells / number of CD8^+^ T cells

Characteristics		N	CD20			CD8		
			High	Low	*p*-Value	High	Low	*p*-Value
Age	< 70	152	108	44		112	40	
	≥ 70	84	69	15	0.055	66	18	0.400
Sex	Male	194	142	52		141	53	
	Female	42	35	7	0.154	37	5	0.047^*a^
History of other cancer	No	204	153	51		155	49	
	Yes	32	24	8	1.000	23	9	0.660
Location	Ce / Ut	21	14	7		13	8	
	Mt	142	111	31		107	35	
	Lt / Ae	73	52	21	0.359	58	15	0.280
cT category	T 1 / 2	111	85	26		75	36	
	T 3 / 4	125	92	33	0.598	103	22	0.008^**^
cN category	N -	88	64	24		57	31	
	N +	148	113	35	0.536	121	27	0.004^**^
cStage category	0 - II	119	89	30		81	38	
	III / IVa	117	88	29	0.940	97	20	0.008^**^
Neoadjuvant Chemotherapy	No	84	62	22		57	27	
	Yes	152	115	37	0.754	121	31	0.047^*^
Myelosuppression grade	≤ 2	131	98	33		105	26	
	3	14	10	4		10	4	
	4	7	7	0	0.130	6	1	0.695
pT category	T 1a / 1b / 2	137	103	34		95	42	
	T 3 / 4a	99	74	25	0.939	83	16	0.009^**^
pN category	N 0	103	77	26		76	27	
	N 1 - 4	133	100	33	0.940	102	31	0.608
pStage category	0 - II	143	107	36		104	39	
	III / IVa	93	70	23	0.939	74	36	0.229
Histological therapeutic effect	0 / 1a	114	86	28		90	24	
	1b / 2	38	29	9	0.913	33	7	0.725
Adjuvant chemotherapy	No	135	106	29		103	32	
	Yes	101	71	30	0.150	75	26	0.719

*Ce* Cervical esophagus, *Ut* Upper thoracic esophagus, *Mt* Middle thoracic esophagus, *Lt* lower thoracic esophagus, *Ae* Abdominal esophagus, *T D*epth of invasion, *N* Nodal status

^*^p < 0.05,

^**^p < 0.01, statistically significant

^a^Fisher’s exact test

### Clinical significance of CD23^+^ TLS (GC-TLS)

**Fig. 3:**
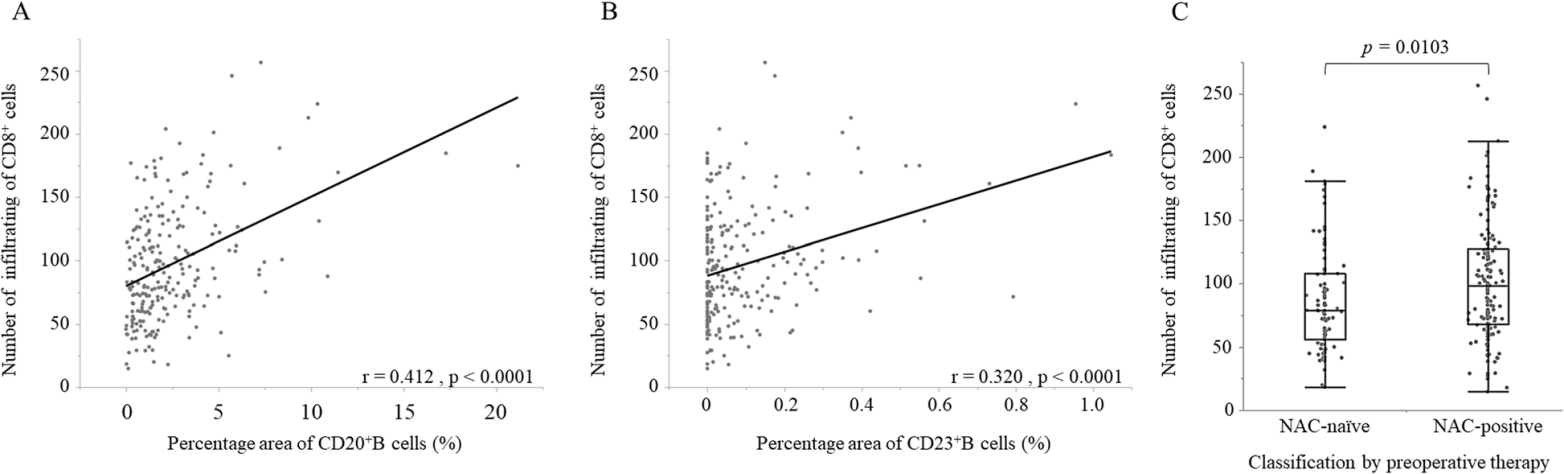
The correlation was analyzed using Spearman’s rank correlation coefficients and Wilcoxon rank sum test. A. The density of CD20^+^ B cells showed a weak positive correlation with the number of CD8^+^ T cells. B. The density of CD23^+^ B cells showed a weak positive correlation with the number of CD8^+^ T cells. C. The number of CD8^+^ T cells was significantly higher in patients with neoadjuvant chemotherapy than in those without neoadjuvant chemotherapy.

The presence of TLSs detected by CD20 alone showed no relationship to the prognosis of patients treated with NAC. B cells usually differentiate from naïve B cells in lymphoid tissues by germinal centers (GC) and are thought to transform into memory B cells. The CD23 expression of GCs in TLSs was examined to evaluate the function of TLS. CD23^+^ cells were aggregated within TLSs and were consistent with the light zone on HE staining (Fig. 1G). All of the aggregates of CD23^+^ cells were in the TLS formation and were not lacking in TLSs. It was previously reported that TLS maturation stage was evaluated, and secondary follicle-like TLS containing lymphocytic aggregates of CD23^+^ cells was indicated as mature TLS [12]. Following these previous studies, a colony of CD23^+^ cells within TLS was defined as a TLS containing germinal center (GC-TLS). The patients were also classified by CD23 into two groups, a GC-TLS-rich group (*n* = 59) and a GC-TLS-poor group (*n* = 177). It was found that GC-TLSs, as well as normal TLS formations, and the infiltration number of CD8^+^ T cells had a weak positive correlation in patients with or without NAC (*r* = 0.412, *p* < 0.0001, *r* = 0.320, *p* < 0.0001, respectively) (Fig. 3A,B). Subsequently, the correlation between GC formation in TLSs and CD8^+^ T cells with or without NAC was examined. Of the 236 patients, the number of patients who received NAC was 152 (64%). The number of infiltrating CD8^+^ T cells was significantly higher in patients with NAC than in those without NAC (*p* = 0.010) (Fig. 3C). Regarding GC formation, patients who underwent NAC tended to have a higher CD23 density (*p* = 0.055) (Table 2).

**Table 2:** Correlations between the clinicopathological factors and density of the CD23^+^ cells

Characteristics		N	CD23		
			High	Low	*p*-Value
Age	< 70	152	39	113	
	≥ 70	84	20	64	0.753
Sex	Male	194	44	150	
	Female	42	15	27	0.086
History of other cancer	No	204	51	153	
	Yes	32	8	24	1.000
Location	Ce / Ut	21	5	16	
	Mt	142	34	108	
	Lt / Ae	73	20	53	0.852
cT category	T 1 / 2	111	28	83	
	T 3 / 4	125	31	94	0.940
cN category	N -	88	22	66	
	N +	148	37	111	1.000
cStage category	0 - II	119	31	88	
	III / IVa	117	28	89	0.707
Neoadjuvant Chemotherapy	No	84	15	69	
	Yes	152	44	108	0.055
Myelosuppression grade	≤ 2	131	35	96	
	3	14	4	11	
	4	7	5	1	0.058
pT category	T 1a / 1b / 2	137	36	101	
	T 3 / 4a	99	23	76	0.593
pN category	N 0	103	27	76	
	N 1 - 4	133	32	101	0.705
pStage category	0 - II	143	39	104	
	III / IVa	93	20	73	0.314
Histological therapeutic effect	0 / 1a	114	32	82	
	1b / 2	38	12	28	0.681
Adjuvant chemotherapy	No	135	38	97	
	Yes	101	21	80	0.194

*Ce* Cervical esophagus, *Ut* Upper thoracic esophagus, *Mt* Middle thoracic esophagus, *Lt* lower thoracic esophagus, *Ae* Abdominal eso-phagus, *T* Depth of invasion, *N* Nodal status

### The differential impact of GC-TLSs on prognosis compared with CD20-TLSs

**Fig. 4:**
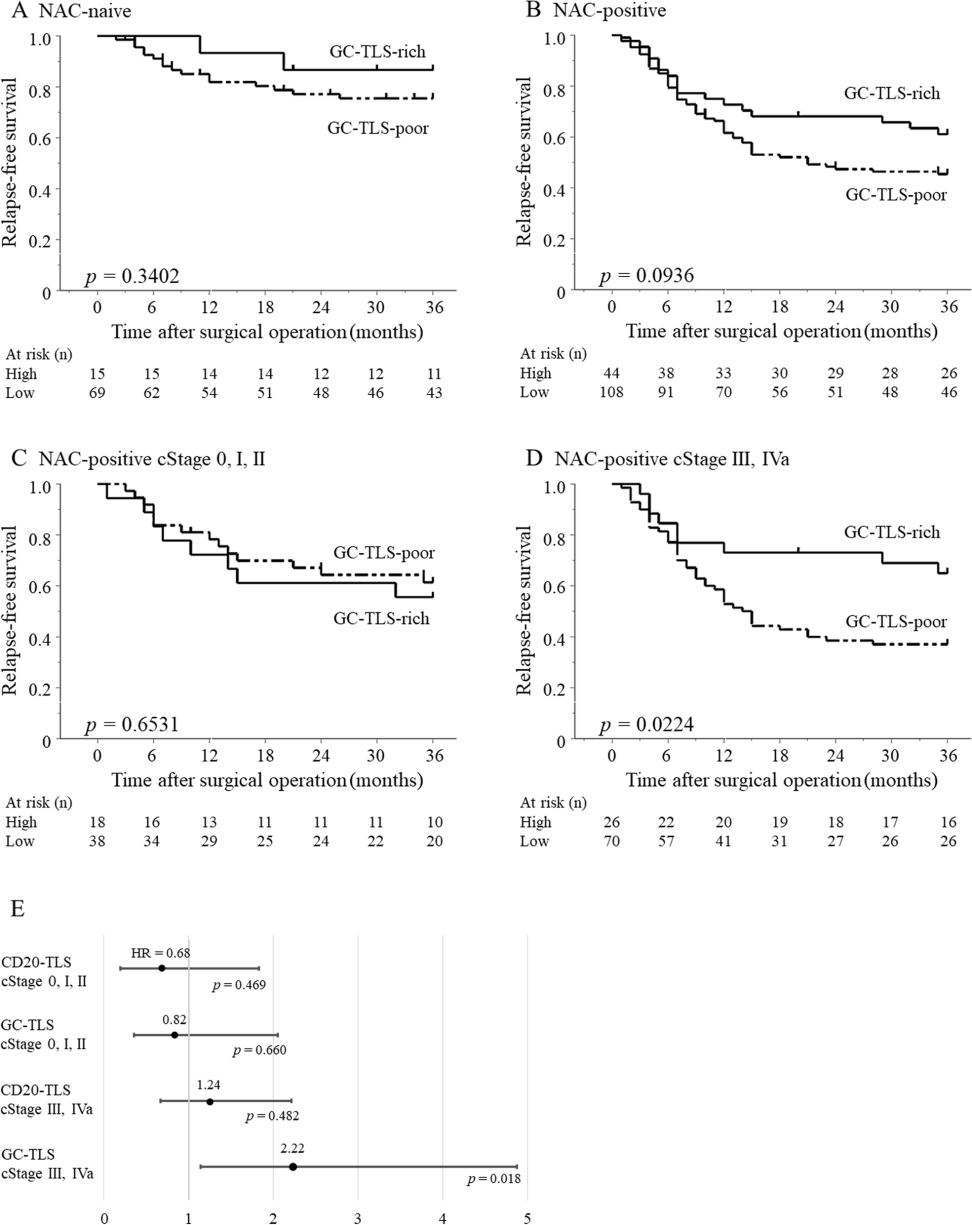
Kaplan–Meier survival curves of the relapse-free survival for GC-TLS formation in patients with ESCC. A. The group who received neoadjuvant chemotherapy (survival rate at 36 months: 86.7%, and 75.5%, respectively). B. The group without neoadjuvant chemotherapy (survival rate at 36 months: 61.1%, and 45.5%, respectively). C. The group who received neoadjuvant chemotherapy with clinical stage 0, I, and II (survival rate at 36 months: 61.4%, and 55.6%, respectively). D. The group who received neoadjuvant chemotherapy with clinical stage III, IVa (survival rate at 36 months: 37.1%, and 65.0%, respectively). E. Hazard ratios were calculated by grouping patients according to clinical progression (III, IVa and 0, I, II) as forest plots. HR, hazard ratio; *p* < 0.05, statistically significant

The prognosis tended to be good in the GC-TLS-rich group in the patients who received NAC, but there was no significant difference in both groups with and without NAC (*p* = 0.340, *p* = 0.094, log-rank test, respectively) (Fig. 4A, B). Regarding the prognosis of the NAC group, more patients had long-term survival in the GC-TLS-rich group than in the GC-TLS-poor group (Fig. 4C). To understand the prognostic value of GC-TLS, subgroup analysis was performed by clinical stage in patients who received NAC. While there was no significant difference in the 3-year survival rate in patients with clinical stage 0, I, or II, the 3-year survival rate of patients with clinical stage III or IVa was significantly better in the GC-TLS-rich group than in the GC-TLS-poor group (*p* = 0.022, log-rank test) (Fig. 4C,D). Hazard ratios were calculated by grouping patients according to clinical progression (III, IVa and 0, I, II). We found that GC-TLS had a significantly greater prognostic impact, in terms of hazard ratio, in comparison to TLS in patients with progression III, IVa (Fig. 4E). Furthermore, GC-TLS was an independent prognostic factor in the study of prognostic factors with the addition of CD8^+^TIL (Table 3).

**Table 3:** Univariate and multivariate analysis of prognostic factors in the cStage III, IVa ESCC treated with NAC

Variable	36 months RFS Univariate		*p*-value	36 months RFS Multivariate		*p*-value
	Hazard ratio	(95% CI)		Hazard ratio	(95% CI)	
CD20 (high/low)	0.804	(0.442–1.462)	0.4744	1.176	(0.602–2.296)	0.6355
CD8 (high/low)	0.664	(0.342–1.291)	0.2276	0.664	(0.325–1.358)	0.2618
CD23 (high/low)	0.450	(0.219–0.922)	0.0292^*^	0.437	(0.206–0.924)	0.0302^*^

*RFS* Relapse free survival, *CI* Confidence interval

^*^p < 0.05, statistically significant

### Correlation of GC-TLSs with the effect of neoadjuvant chemotherapy

**Fig. 5:**
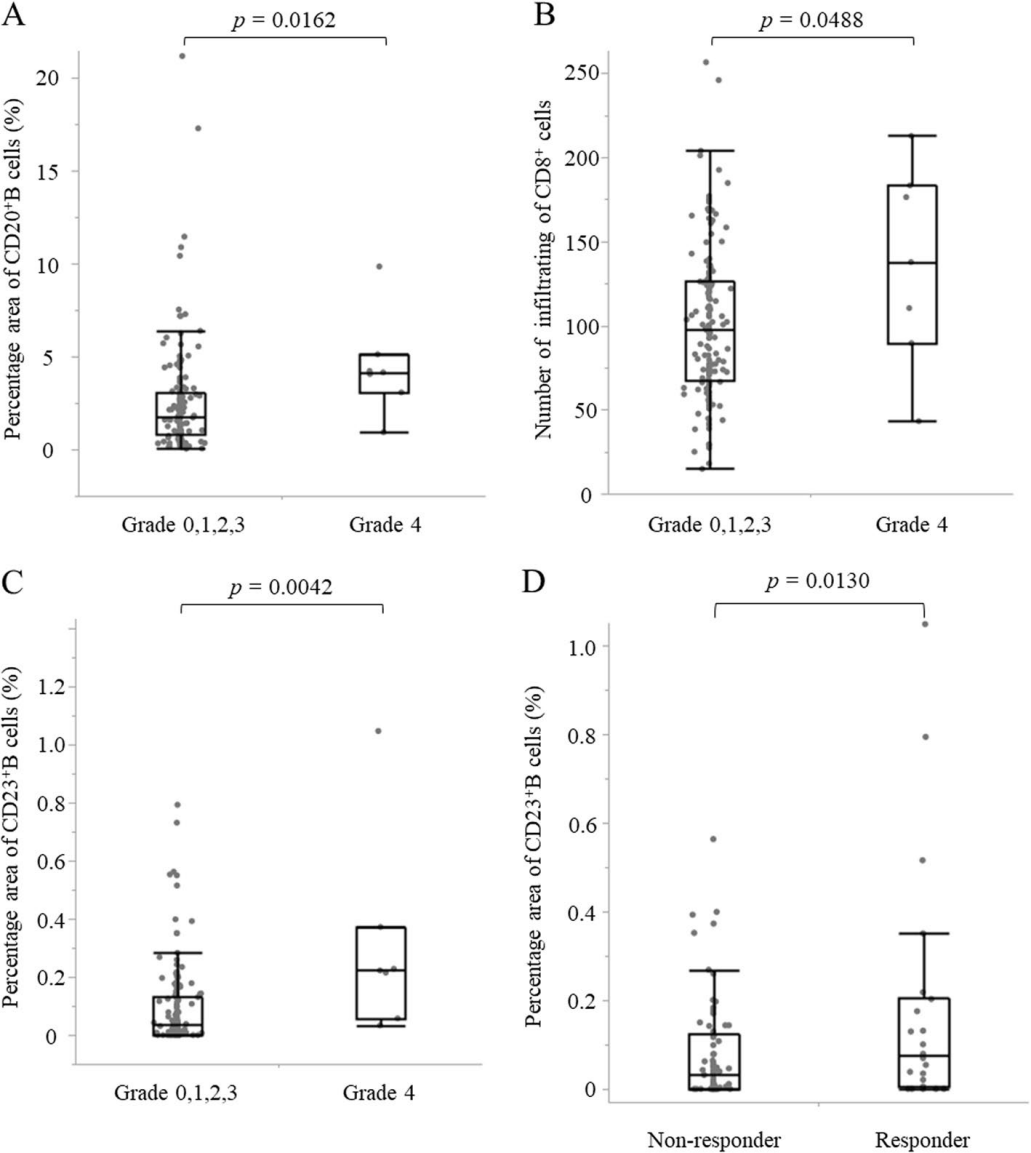
Correlation with GC-TLSs and effect of neoadjuvant chemotherapy (A. CD20^+^ B cells, B. CD8^+^ T cells, C. CD23^+^ B cells). D. The percentage area of CD23^+^ B cell agglutination in 96 locally advanced cases

**Fig. 6:**
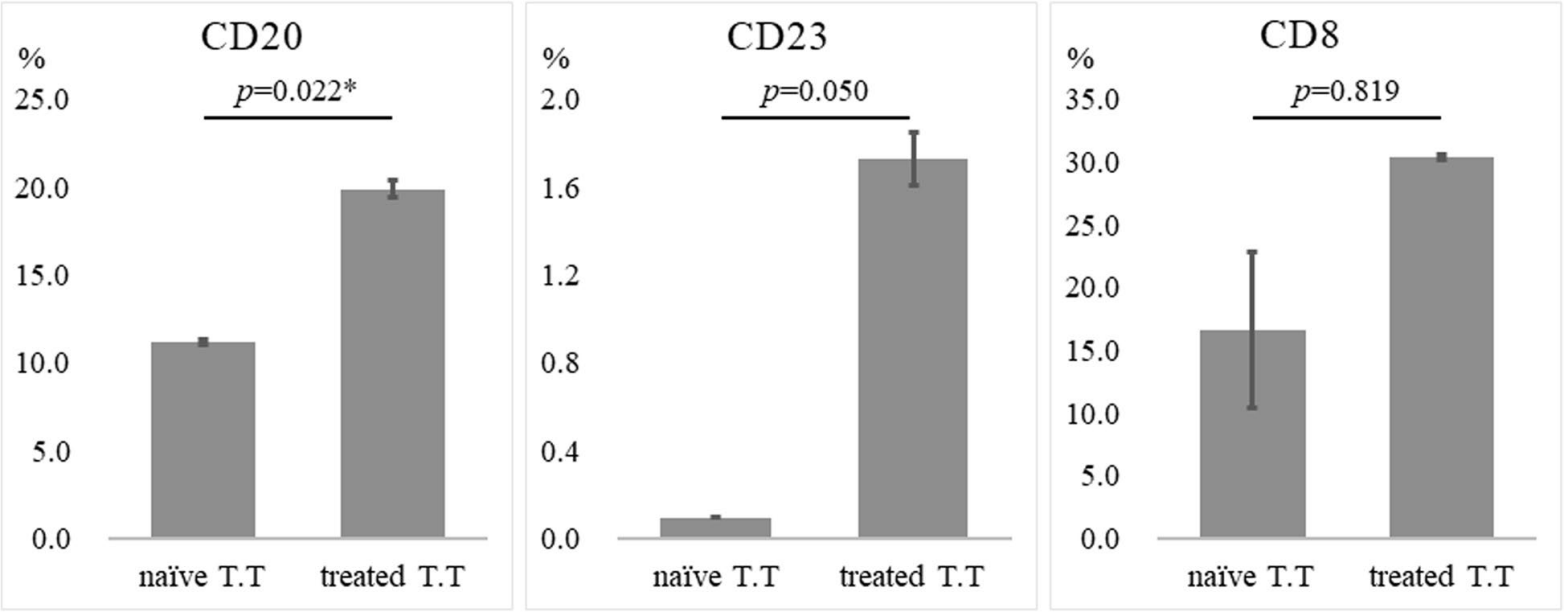
Flow cytometry analysis of the B cell phenotype in PBMCs co-cultured with cell line (T.T). The mean ± standard error of the proportion of the co-cultured CD20^+^, CD23^+^ and CD8^+^ cells with naïve (*n* = 7) or treated (*n* = 3) T.T. *p < 0.05, statistically significant

**Fig. 7:**
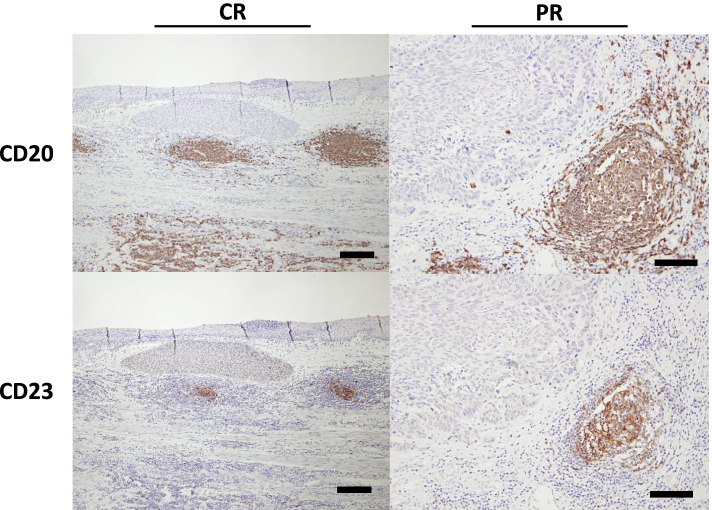
Representative immunohistochemistry of the primary lesions of patients who underwent ICI treatment for postoperative of ESCC and showed a clinical response (PR, CR). Scale bars, CR case 200 µm, PR case 100 µm

Grade 3 or greater myelosuppression events were observed in 21 (13.8%) of 152 patients undergoing NAC. Postoperative adjuvant chemotherapy was given to 101 patients (43%). The relationship between myelosuppression that developed during NAC and immune cells was investigated. Myelosuppression involved three strains of cytopenia in this study. The patients with Grade 4 myelosuppression had significantly greater density of TLSs and GCs and infiltration of CD8^+^ T cells than other patients (*p*=0.016, *p*=0.004, *p*=0.049, respectively) (Fig. 5). In addition, to assess the association between chemotherapy and TLS maturation at the tumor site, the GCs of patients with and without chemotherapy in locally advanced cases were compared. GC formation was compared by classifying 96 patients with locally advanced cT3 or deeper. A responder was defined as a patient diagnosed as locally advanced cancer of T3 or deeper from preoperative examination and as T1 on postoperative pathology due to the effect of NAC. The responder group had better GC formation than the non-responder group (*p*=0.013) (Fig. 5D). Co-culturing of PBMCs with an anticancer drug-treated esophageal squamous cell carcinoma cell line increased the CD20 and CD23 expression in PBMCs *in vitro* (Fig. 6). GC-TLS was observed in abundance in the primary lesions of patients who underwent ICI treatment for postoperative recurrence of ESCC and who showed a clinical response (PR, CR) (Fig. 7).

## Discussion

This study showed that the presence of TLSs with germinal center formation around the primary tumor (GC-TLS) was an important prognostic factor in post-NAC specimens of ESCC. In cases where GC-TLSs were frequently observed, the clinical efficacy of NAC was high, and long-term prognosis was achieved even in locally advanced esophageal cancer such as clinical stages 3 and 4. This indicates that the change in the local immune environment of esophageal cancer by NAC is important for prognosis, and it suggests that the presence of GC-TLSs may be used to determine the indications for immunotherapy in the future.

In the present study, it was found that the CD20^+^ cells that were present in ESCC tissue were often present as an aggregated form. Within this cluster, follicular DCs and T cells were also present and were identified as TLS. And the high-TLS group had a better prognosis in early-stage esophageal cancer, but there was no difference in cases with an advanced stage of 3 or more. The reason for this may be the increased number of Treg cells and M2 macrophages that act as immunosuppressors around TLSs or locally in the tumor. For example, in mouse experiments, TLSs increased after suppressing Tregs in TLSs, and, at the same time, CD4 and CD8 cells increased [13]. Further investigations are required, such as of cytokine production ability of macrophages within cancer-associated TLSs, or activation and proliferation status of T and B cells in TLSs with a higher ratio of tingle-body macrophages [14]. The present study focused on the maturation of TLSs, i.e., the formation of the germinal center, because CD20 alone is not sufficient to assess functional TLSs. B cells in the germinal center express CD23 and differentiate into plasma cells and memory B cells. According to Barros et al., B cells showed overgrowth clonality in GC-TLS-rich tumor regions [15]. This suggests that the co-localization of B cells and T cells in TLSs causes antigen presentation in the tumor microenvironment.

In the present study, the prognosis of patients with a high density of matured GC-TLSs in stages 3 and 4 was good. Furthermore, the density of GC-TLSs was higher in patients who underwent NAC and achieved a clinical response. Importantly, there was no association between the prognosis of patients with NAC and the density of CD20-TLSs. Silina reported that the presence of TLSs was not a prognostic factor in the group receiving preoperative chemotherapy for lung cancer [12]. They showed that, in patients treated with NAC, TLS density was similar, but GC formation was impaired, and the prognostic value of TLS density was lost because of corticosteroids during chemotherapy. Wu also reported that the tumor immune environment is lost with repeated 5-FU, so that long-term chemotherapy can impair tumor immunity [16]. On the other hand, by chemotherapy-induced complement activation, ICOSL on B cells were induced, eliciting the T cell antitumor effect in mice [17]. The present results suggested that chemotherapy may mature B cells and lead to the development of GC-TLSs. Moreover, it was shown that GC-TLS formation tended to increase in cases of myelosuppression during chemotherapy. Neutrophils are markedly reduced in myelosuppression. We have previously reported that local neutrophil infiltration in gastric cancer is associated with an immunosuppressive environment [18]. Therefore, we hypothesized that myelosuppression also reduces local neutrophils, alleviating the immunosuppressive environment and promoting B cell maturation.

Thus, the present results suggest that chemotherapy-induced B-cell maturation and formation of TLSs with a germinal center (GC-TLSs) may result in an anti-tumor immune response and a good long-term prognosis treated with NAC. Recently, there have been reports on the relationship between the maturation of TLSs and the effectiveness of ICI treatment. For example, Helmink et al. performed RNA-seq of tissues from patients with melanoma and renal cell carcinoma who participated in clinical trials of immune checkpoint inhibition therapy and reported that the expressions of genes involved in changes in B cell function and TLS density were markedly increased in the treatment response group, and there were increases in memory B cells and germinal center-like B cells [19]. The fact that GC-TLS was observed more frequently in patients who showed clinical response to ICI treatment in our study suggests a relationship between ICI treatment response and B cells, i.e., TLS. Thus, we believe that the phenotype of TLSs is important in assessing their functionality.

There are several limitations in this study. First, it was not possible to show direct evidence that GC-TLSs are induced by chemotherapy. As we previously reported, chemotherapy induces maturation of dendritic cells in ESCC carcinoma tissues, and we suggest that it probably induces B cell differentiation as well. Second, it was not possible to analyze the subsets of B cells in the GC-TLSs, but we have previously shown that plasma cells, germinal center B cells, and memory B cells are more abundant in the TLSs of gastric cancer tissues than in non-cancerous areas, suggesting that B cell differentiation is probably advanced in GC-TLSs. Furthermore, the number of cases studied was small because this was a single-center, retrospective study. As a future issue, it is necessary to clarify the mechanism of chemotherapy-induced TLS maturation, such as the effect of chemotherapy regimen and dosage on the development of TLSs, by accumulating a large number of cases. Previously, Li et al. defined mature TLS morphologically as the presence of CD21 follicular dendritic cells and high endothelial cells within the TLS in SCC of the head and neck, which is histologically similar to ESCC, but the present study was considered novel in that it defined mature TLS functionally by CD23 [20].

## Conclusion

TLS maturation (GC-TLSs) may be important in the evaluation of local tumor immune response in patients who underwent NAC in esophageal cancer. The present results suggest that maturation of TLSs may be a target for predicting the efficacy of immunotherapy and treatment of ESCC.

## Data Availability

The datasets used and analyzed during this study are available from the corresponding author on reasonable request, and most of the original data are included within the article.

## Abbreviations

5-FU: 5-Fluorouracil
CD: Cluster of differentiation
CDDP: Cis-dichlorodiammine platinum
CT: Computed tomography
DAB: 3–3’-Diaminobenzidine
ESCC: Esophageal squamous cell carcinoma
FFPE: Formalin-fixed paraffin-embedded
GC: Germinal center
HE: Hematoxylin and eosin
HEV: High endothelial venule
ICI: Immune checkpoint inhibitor
ICOSL: Inducible T-cell co-stimulator ligand
NAC: Neoadjuvant chemotherapy
PBMC: Peripheral blood mononuclear cell
PNAd: Peripheral node addressin
RFS: Relapse-free survival
RNA: Ribonucleic acid
TIL: Tumor infiltrating lymphocyte
TLS: Tertiary lymphoid structure
